# Differential Behavioral Pathways Linking Personality to Leadership Emergence and Effectiveness in Groups

**DOI:** 10.1177/01461672241246388

**Published:** 2024-04-24

**Authors:** Tobias M. Härtel, Felix Hoch, Mitja D. Back

**Affiliations:** 1 Osnabrück University, Germany; 2 University of Münster, Germany

**Keywords:** Big five personality traits, leadership emergence, leadership effectiveness, behavioral processes, interpersonal perception

## Abstract

This study integrates leadership process models with process models of personality and behavioral personality science to examine the behavioral–perceptual pathways that explain interpersonal personality traits’ divergent relation to group leadership evaluations. We applied data from an online group interaction study (*N* = 364) alternately assigning participants as leaders conducting brief tasks. We used four variable types to build the pathways in multiple mediator models: (a) Self-reported personality traits, (b) video recordings of expressed interpersonal behaviors coded by 6 trained raters, (c) interpersonal impressions, and (d) mutual evaluations of leadership emergence/effectiveness. We find interpersonal big five traits to differently relate to the two leadership outcomes via the behavioral-perceptual pathways: Extraversion was more important to leadership emergence due to impressions of assertiveness evoked by task-focused behavior being strongly valued. Agreeableness/emotional stability were more important to leadership effectiveness due to impressions of trustworthiness/calmness evoked by member-focused/calm behavior being stronger valued.

The performance and satisfaction of (work) groups crucially depend on the person who emerges as a group leader and how effectively they lead the group (e.g., Burke et al., 2006; Zaccaro et al., 2001). Also, group members’ evaluative perceptions of leadership emergence (becoming influential in a group) and effectiveness (performing effectively in the leader role) shape their willingness to contribute and collaborate, thus impacting group functioning and cohesiveness (e.g., Hogg, 2001; Meindl, 1995). Thereby, an individual’s personality is a decisive predictor of both evaluations of leadership emergence and leadership effectiveness in social groups (e.g., Badura et al., 2022; DeRue et al., 2011). Furthermore, personality traits often relate differently to the two leadership outcomes (e.g., Judge et al., 2002), suggesting that the naturally emerging group leader may not be the most effective.

While extensive research shows that personality traits affect evaluations of leadership emergence and effectiveness and that these effects can be distinct, our understanding of the explanatory behavioral and perceptual processes is still evolving (Blake et al., 2022; DeRue et al., 2011; Hu et al., 2019). We open this black box between personality and leadership outcomes to explain the underlying causal mechanisms by marrying leadership process models (e.g., Antonakis et al., 2012; Zaccaro et al., 2018) with process models of personality (e.g., Back, 2021; Back et al., 2023). Who emerges as a group leader and who performs effectively in this role represent inherent interpersonal questions. In this study, we thus illuminate the *interpersonal* leadership domain by focusing on the three clearly distinguishable key overt interpersonal behavioral factors (task-focus, member-focus, and calmness) identified in behavioral personality science (Breil et al., 2021, 2022; Leising & Bleidorn, 2011) and the leadership literature (e.g., Bass, 1990; Yukl, 2012). These interpersonal behaviors are proposed to evoke leadership-relevant interpersonal impressions (assertive, trustworthy, and calm) that in turn should be differently evaluated regarding leadership emergence versus effectiveness. This way, we provide a behavioral–perceptual explanatory model unraveling the divergent main effects of interpersonal personality traits (extraversion, agreeableness, and emotional stability) on evaluations of leadership emergence and effectiveness (see Figure 1).

**Figure 1.**
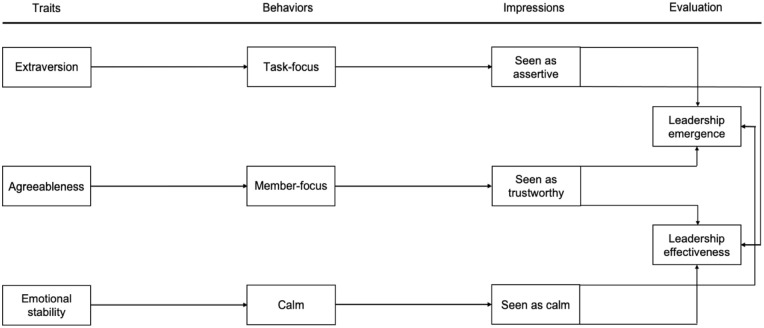
A Behavioral Pathway Approach Linking Personality to Leadership Outcomes in Groups.

The present study contributes to ongoing discussions in the leadership literature on several fronts. While the main effects of personality traits on leadership outcomes are well-established (Judge et al., 2002), the why/how of the underlying behavioral–perceptual mechanisms remains much less understood. Leadership refers to “what people do to influence others” (Fischer et al., 2023, p. 1) and thus manifests in behavioral leader-follower dynamics. Yet, truly behavioral constructs are “almost completely absent” in leadership research (Fischer et al., 2023, p. 1; see also Banks et al., 2021). In this study, we make a clear distinction between the behaviors evoked by personality traits (i.e., what leaders actually do) and the mutual impressions and evaluations formed by interaction partners. This way, we deepen our process understanding of the nuanced interplay between personality, behaviors, and impressions and provide a more comprehensive explanation of the phenomena, enhancing the generalizability of findings and assessment of boundary conditions (Fischer et al., 2017). We thereby answer specific calls in the interpersonal leadership domain to “more directly measure individual behavior and peer perceptions” for extraversion (Hu et al., 2019), to “attend to the questions of ‘why’ and ‘how’ leadership agreeableness influences relevant outcomes” (Blake et al., 2022, p. 13), and to “specify mechanisms that explain the effect of leader traits such as emotional stability, which influence effectiveness” (DeRue et al., 2011, p. 40). Finally, we break new ground by comparing the behavioral–perceptual mediating processes for the distinct constructs of leadership emergence and effectiveness to “reveal important similarities as well as differences” (Marinova et al., 2012, p. 1270).

## A Behavioral Pathway Approach Linking Personality to Leadership Outcomes in Groups

According to process models of personality (e.g., Back, 2021; Back et al., 2011, 2023; Grosz et al., 2020; Nestler & Back, 2013), expressed behaviors that evoke interpersonal impressions take the key role in explaining the mechanisms linking personality traits to social outcomes. More specifically, personality traits are latent constructs that are not directly observable in interpersonal interactions. Thus, conceptually, personality cannot affect social outcomes directly but only indirectly. Thereby, the necessary prerequisite for an impact on social outcomes is that personality traits must be expressed in more proximal observable behavior (behavioral expression). Furthermore, the sufficient prerequisite is that these expressed behaviors are then observed and formed to even more proximal impressions by interaction partners (interpersonal perception), which, in turn, are evaluated regarding social outcomes, like leadership emergence and effectiveness (evaluation). For example, Härtel et al. (2021) utilized a behavioral pathway approach to investigate how the personality trait narcissism (entitled self-importance) is linked with leadership emergence in social groups (see also Leckelt et al., 2015): Narcissists’ agentic components were expressed through dominant-expressive behaviors, which evoked impressions of being seen as assertive, which in turn led to narcissists’ leadership emergence. Taking such a behavioral pathway approach also seems promising for understanding how interpersonal big five traits affect evaluations of leadership emergence and effectiveness. Here, process models of personality can serve as leadership process models (e.g., Antonakis et al., 2012; Zaccaro et al., 2018) examining more proximal behaviors and impressions as the causal chain between distal determinants (personality traits) and leadership outcomes.

### Core Interpersonal Behaviors

Behavioral personality science reveals the key overt behaviors that occur and can be reliably observed in interpersonal group constellations, which are at the core of the inherent interpersonal questions of who emerges as a group leader and who performs effectively. Specifically, we build on a three-dimensional underlying structure of interpersonal behaviors (agency, communion, calmness) which has recently been conceptually and empirically demonstrated (Breil et al., 2021, 2022; Leising & Bleidorn, 2011).

Expanding upon the interpersonal circumplex model (Wiggins, 1979), interpersonal theory posits two fundamental behavioral dimensions that form the basis for describing observable behaviors in interpersonal situations: *Agency* (getting ahead/dominance/competence/assertiveness) referring to task functioning and goal achievement versus *communion* (getting along/affiliation/warmth/trustworthiness) referring to functioning in social relations (e.g., Dawood et al., 2018; Hogan & Holland, 2003; Hopwood, 2018; Wojciszke et al., 2009). These dimensions have also emerged as fundamental behavioral dimensions in the leadership literature labeled as *task-focus* (task-orientation/initiating structure/production-centered) corresponding with agency and *member-focus* (member-orientation/consideration/employee-centered) corresponding with communion (e.g., Bass, 1990; Yukl, 2012). Task- and member-focus are key proximal behavioral dimensions predicting leadership outcomes (e.g., Burke et al., 2006; DeRue et al., 2011) and have been repeatedly suggested as promising mediators between personality and leadership outcomes (e.g., Judge et al., 2004, 2009).

Besides agency and communion, behavioral personality science affirms interpersonal behavior related to the display of *interpersonal calmness* (as the opposite pole of interpersonal nervousness and social anxiety; Borkenau et al., 2004; Naumann et al., 2009). Specifically, differences in interpersonal calm behaviors such as coping well with stress, handling emotions, and responding in a relaxed way (e.g., stable vs. unstable, robust vs. vulnerable, relaxed vs. tense) have been suggested to represent a third distinct fundamental dimension of interpersonal behavior that does emerge and can be observed reliably across a variety of everyday interpersonal situations (Leising & Bleidorn, 2011). This indicates that interpersonal calm behaviors are more observable and universally relevant to interpersonal dynamics than often assumed, complementing the agency/communion dimensions.

Recently, interpersonal calm behaviors have also shown to play a central role in evaluating interpersonal behavior in personnel selection settings (Breil et al., 2021, 2022), further demonstrating its stability across social situations including work contexts. As mutual evaluations of leadership potentials within social groups pose an inherent interpersonal question, exploring this third basic meaning dimension of interpersonal behavior may help to encapsulate a fuller range of a leader’s interpersonal behavior and thus contribute to a more comprehensive understanding of the interpersonal leadership domain. This way, we aim to shed some light on the behavioral process mechanisms behind a growing body of evidence suggesting that calmness-related traits, behaviors, and impressions are instrumental in leadership contexts (e.g., Bowman, 2022; Hartmann et al., 2020; Ishaq et al., 2021; King et al., 2016; Klus & Müller, 2021; Li et al., 2012; Ormiston et al., 2022).

### Core Interpersonal Personality Traits

In the present study, we illuminate the interpersonal leadership domain by building on the three-dimensional structure of observable interpersonal behaviors in social group settings. Interindividual differences in the three major meaning dimensions of observable interpersonal behavior refer to unique patterns of what individuals tend to do, and thus, mirror enduring differences in interpersonal personality traits. The focal personality traits of the present study therefore follow directly from the conceptual choice to focus on the basic interpersonal behaviors informed by recent advances in behavioral personality science (Breil et al., 2021, 2022; Leising & Bleidorn, 2011). In particular, task-focus and member-focus have been shown to uniquely match (e.g., Barford et al., 2015; Leising & Bleidorn, 2011) the personality traits of extraversion (e.g., assertive, active, and energetic) and agreeableness (e.g., sympathetic, kind, and warm), representing the two most interpersonal big five traits (McCrae & John, 1992). Complementing this picture, emotional stability (e.g., calm, relaxed, and stable) corresponds to interpersonal calm behavior (Leising & Bleidorn, 2011).

As a consequence of this conceptual decision, the remaining big five traits of conscientiousness and openness are not in the spotlight of this study that employs a framework tailored to social group constellations seeking to illuminate the interpersonal leadership domain. Of course, conscientiousness and openness also have substantial impacts on leadership outcomes (e.g., Judge et al., 2002), and we have only just begun to understand the underlying behavioral–perceptual mechanisms (see Marinova et al., 2012). Yet, both traits have been demonstrated to be less expressed and less openly observable in interpersonal group constellations—they have been found to be barely reflected in interpersonal behavior information and were accordingly labeled the “*least* interpersonal of the big five factors” (Leising & Bleidorn, 2011, p. 990).^1^ Following a behavioral personality science perspective, these traits may be assumed to be less in focus when it comes to the inherently interpersonal phenomenon of who emerges and performs effectively as a group leader. Future research may extent the scope beyond the interpersonal domain and adapt the behavioral pathway approach by building on promising behavioral dimensions like competent behavior (e.g., Breil et al., 2022) for conscientiousness or change-oriented behavior (e.g., DeRue et al., 2011) for openness.

### Core Interpersonal Impressions

Expressed interpersonal behaviors evoke interpersonal impressions that are evaluated by group members regarding leadership outcomes. Being seen as assertive represents the interpersonal impression at the core of agency (e.g., Abele et al., 2008; Abele & Wojciszke, 2007), which has also been considered in previous initial research examining agentic pathways from personality to leadership (Härtel et al., 2021; Hu et al., 2019), and which should be central for leadership evaluations (e.g., Ames & Flynn, 2007). Likewise, being seen as trustworthy represents the interpersonal impression at the core of communion (e.g., Abele & Wojciszke, 2007), which has also been considered in initial studies examining communal pathways from personality to leadership (Härtel et al., 2021; Marinova et al., 2012), and which should be central for leadership evaluations (e.g., Ferrin & Dirks, 2002; Legood et al., 2021). Finally, being seen as calm can be construed as the core interpersonal impression evoked by interpersonal calm behaviors (Breil et al., 2022; Leising & Bleidorn, 2011). While calmness is not yet well embedded in the leadership literature, there are initial hints that calmness may be important to leadership evaluations (e.g., Ennis et al., 2015; Klus & Müller, 2021; Silard & Dasborough, 2021).

**Hypothesis 1a (b/c):** Extraversion (agreeableness/emotional stability) has an indirect effect on leadership outcomes that is mediated by rated task-focused (member-focused/calm) behavior and interpersonal impressions of being seen as assertive (trustworthy/calm).

## Distinct Effects of Behavioral Pathways Linking Personality With Leadership Emergence and Effectiveness in Groups

A crucial advancement in studying personality effects on leadership outcomes has been distinguishing between two conceptually distinct constructs: Perceptions of leadership emergence versus leadership effectiveness (Judge et al., 2002). Leadership emergence refers to an individual becoming influential in a group, and thus, refers to processes of appearing leaderlike, assuming responsibility, and taking the leadership role (Badura et al., 2022; Hanna et al., 2021). In comparison, leadership effectiveness involves perceptions of an individual’s actual performance in the leadership role, and thus, refers to processes of effectively directing the group toward goals and satisfaction (DeRue et al., 2011; Judge et al., 2002).

Given that perceptions of leadership emergence and leadership effectiveness are distinct conceptual constructs, the traits, associated behaviors, and impressions instrumental in evoking these two basic types of leadership evaluations naturally differ. Following the agency/communion-framework of interpersonal theory (e.g., Bakan, 1966; Dawood et al., 2018; Wojciszke et al., 2009), leadership emergence pertains more clearly to agentic goals like getting ahead of others through individual status achievement, and thus agentic traits like extraversion (as well as the associated behaviors and impressions) may be deemed more influential. In comparison, leadership effectiveness also complements more communal goals like getting along with others by ensuring group member satisfaction, where communal traits like agreeableness (as well as the associated behaviors and impressions) may gain importance. This aligns with prototypical leader theories (e.g., Lord et al., 1984), according to which group members intuitively decide on who will emerge as their group leader by comparing all group members to an inner image of the prototypical leader. Indeed, in prototypical leadership theories, the emphasis often leans toward agentic leader traits over communal ones when envisioning the prototypical leader (Epitropaki & Martin, 2004; Offermann et al., 1994; see also Reichard et al., 2011). Traits associated with calmness are typically not specified in prototypical leadership theories, suggesting that they may not be initially salient as group members emerge into leadership roles. However, when evaluating effective leadership, it may become apparent that interpersonal calmness can promote effective team dynamics, including aspects such as fostering open and calm interpersonal communication, making deliberate and thoughtful rather than rushed and hasty decisions, and handling difficult interpersonal conversations, tensions, or conflicts with composure.

Indeed, empirical findings support the idea that personality traits differently relate to perceptions of leadership emergence versus effectiveness. Extraversion is the trait most consistently associated with leadership emergence (e.g., Ensari et al., 2011; Reichard et al., 2011) and related constructs such as status attainment (e.g., Anderson et al., 2001; Grosz et al., 2020). While extraversion has also been shown to predict leadership effectiveness (e.g., DeRue et al., 2011), it seems to be more important for emerging as a leader than leading effectively (Judge et al., 2002). In contrast, whiles agreeableness has been shown to be relatively unimportant to leadership emergence (e.g., Badura et al., 2022; Ensari et al., 2011; Reichard et al., 2011) and status attainment (e.g., Anderson et al., 2001), it appears to be decisive for leadership effectiveness (e.g., DeRue et al., 2011; Judge et al., 2002). Similarly, emotional stability showed inconsistent and low associations with leadership emergence (e.g., Badura et al., 2022; Ensari et al., 2011; Lord et al., 1986; Reichard et al., 2011; cf. Judge et al., 2002) and status attainment (e.g., Anderson et al., 2001) but was consistently found to be important for leadership effectiveness (e.g., DeRue et al., 2011; Hoffman et al., 2011; Judge et al., 2002). Behavioral pathways could be the key to better understanding why personality traits show distinct main effects on evaluations of leadership emergence and effectiveness by zooming-in on the personality-evoked behaviors and impressions that may be differently evaluated in terms of the two leadership outcomes (see also Härtel et al., 2021, for a similar approach focusing on leadership emergence and popularity).

**Hypothesis 2a:** The indirect effect of the pathway of extraversion, rated task-focused behavior, and being seen as assertive is more positive for evaluations of leadership emergence than leadership effectiveness.
**Hypothesis 2b:** The indirect effect of the pathway of agreeableness, rated member-focused behavior, and being seen as trustworthy is more positive for evaluations of leadership effectiveness than leadership emergence.
**Hypothesis 2c:** The indirect effect of the pathway of emotional stability, rated calm behavior, and being seen as calm is more positive for evaluations of leadership effectiveness than leadership emergence.

## Method

We describe our sampling, data exclusions, manipulations, and all measures used in the study. A codebook (providing an overview of the procedure, materials, and all variables assessed), data, analysis code, and supplemental results are available at https://osf.io/6s9uf/. This study was not preregistered.

### Sample

The sample included 364 participants recruited via social media, e-mail newsletters, advertising posters, and lecture announcements at two German Universities. Participants were compensated with a fixed amount of 21€ and a variable share of up to 9€ based on their performance in the group tasks. Students could choose to substitute the fixed compensation with course credit. All participants could opt to receive feedback on how well they adopted the leaders instructions during the experiment.

The average age was 24.03 (*SD* = 4.00) with most participants being students (86.81%) from various subjects (47.46% business/economics). All 364 participants (218 women) provided complete self-reported personality traits and attended the online Zoom meeting. Initially, there were 368 participants, but we removed four participants who formed a group because one of them lacked German skills, severely impairing group processes.

This sample size surpasses those in similar studies investigating group interactions and mediation models between personality traits, behavioral measures, and social consequences (*N* = 191, Cheng et al., 2013; *N* = 311, Härtel et al., 2021; *N* = 68, Küfner et al., 2013; *N* = 311, Leckelt et al., 2015; *N* = 191, Witkower et al., 2020). These prior studies successfully identified behavioral pathways linking personality to social group outcomes, suggesting our study is sufficiently powered to detect the effects of interest. Hence, we did not conduct a power analysis before data collection. Yet, for a more detailed understanding of power, we applied Schoemann et al.’s (2017) online tool to compute the power for the indirect effect of agreeableness on perceived leadership effectiveness via member-focused behavior and being seen as trustworthy (Hypothesis 1b). We focused on the agreeableness-leadership effectiveness pathway because we anticipated smaller effects compared with extraversion pathways, leading to a more conservative power estimation. Compared with the emotional stability pathways, the required correlations for power estimation have been more thoroughly examined in the literature, enabling a more reliable power estimation. We conducted Monte Carlo power analysis simulations and tested the indirect effects with bootstrapped confidence intervals. The model included two serial mediators, 5,000 replications, and 20,000 Monte Carlo draws per replication while maintaining a 95% confidence level (random seed = 1,234). We conservatively entered a sample size of *n* = 359, considering only complete cases. We conservatively used raw correlation coefficients from prior research instead of corrected ones (*r*_agreeableness, member-focus_ = .26, DeRue et al., 2011; *r*_agreeableness, seen as trustworthy_ = .15, Stavrova et al., 2023; *r*_agreeableness, leadership effectiveness_ = .21, Judge et al., 2002; *r*_member-focus, seen as trustworthy_ = .23, Härtel et al., 2021; *r*_member-focus, leadership effectiveness_ = .39, Judge et al., 2004; *r*_seen as trustworthy, leadership effectiveness_ = .26, Legood et al., 2021). All variables were assumed to be standardized. The computed power was .90.

### Procedure

The study consisted of two parts. First, participants completed an online questionnaire collecting demographic information and self-reported personality traits. Then, participants attended a 2-3 hour online Zoom meeting. For the online meetings, the sample was divided into 79 groups of four to five participants (*M* = 4.61) with 66 mixed-sex groups and 13 same-sex groups (ten female-only). A group size of four to five is consistent with the size of effective working groups (Stangor, 2015), facilitates the identification of leaders (Hare, 1976), and enables all group members to participate in the discussion (Hare, 1981). Participants used their personal technical equipment—a computer with a webcam and microphone. In each session, we assessed two groups simultaneously.

The online meetings started with all participants of the two respective groups. Participants were assigned gender-neutral code names (Van Fleet & Atwater, 1997). We standardized the Zoom settings and ensured that all participants could see each other. Participants were then asked to briefly introduce themselves. Next, participants were randomly assigned to their respective groups, and the two groups were transferred to separate break-out sessions, each with its own experimenter. During the meeting, participants completed an online questionnaire that provided instructions^2^ and assessed perception ratings on interpersonal impressions and leadership evaluations.

Aligned with the group size, participants completed four or five rounds of variations of the Lost on the Moon task (Bottger, 1984; Hall & Watson, 1970; Robins & Beer, 2001). In this task, group members imagined themselves crash-landed on the moon and were asked to rank 15 items according to their importance for group survival. The other tasks create similar settings, where the group members imagine themselves as survivors of plane crashes in the desert or an arctic environment, lost at sea after a maritime accident, or colonists in the 18th century plagued by drought and disease. Each round comprised a 5-min period for individual ranking of the 15 items, followed by up to 15 min for group discussion.

Each participant assumed the group leader role once, with the task order randomized. The assigned leader^3^ shared their screen with a template to rank the items and was made responsible for guiding the discussion and submitting the group ranking. The experimenter ensured that all group members could see the leader’s shared screen, video, and the videos of all other members. Following the group ranking submission, all group members individually evaluated the leader for that round, whereby the leader responded to the same items as self-evaluations.

### Measures

#### Personality

We measured extraversion (α = .87), agreeableness (α = .82), and emotional stability (α = .89) as self-reports based on the Big Five Inventory–2 (BFI-2, Soto & John, 2017) in its German translation by Danner et al. (2019). The three traits were assessed with 12 items each using five-point scales ranging from 1 (*do not agree at all*) to 6 (*agree completely*).

#### Behavioral Ratings

The behavioral ratings of task-focus, member-focus, and calm behavior were based on the video recordings during the Zoom group discussions. The video footage included (a) self-directed webcam recordings of the leader (target person of the ratings), (b) the leader’s shared screen displaying the group ranking template, and (c) self-directed webcam recordings of the other group members. The ratings were conducted on six-point scales ranging from 1 (*not at all*) to 6 (*very strongly*). Six^4^ raters (four women), blind to the purpose of the study, independently viewed the recordings^5^ in randomized orders and made their ratings after each viewing. The raters were business or psychology students who conducted the coding as part of their employment as student assistants or research interns.

Raters underwent comprehensive training to establish a shared understanding of the behaviors and to make use of the full-scale range ensuring reliable and valid assessments. The training was based on the recommendations of Grünberg et al. (2018) comprising four steps. First, the authors analyzed the recordings to select five leaders showcasing varying levels of behavioral ratings. Second, raters attended a training session featuring a lecture on the behaviors to be rated and rater biases. Subsequently, the raters independently rated the behaviors in the five sample recordings. Third, these behavioral ratings were compared with the authors’ behavioral ratings and disagreement was discussed in a second training session. Fourth, additional sample recordings were jointly viewed to align the behavioral ratings.

Behaviors were rated on the meso-level (circumscribed behavioral expressions), positioned between global labels (macro-level), and counting micro-behaviors (micro-level), ensuring both reliable and psychologically meaningful ratings (Funder et al., 2000). Raters used sheets with predefined behavioral labels, accompanied by explanations and examples of associated behaviors tailored to the specific interaction task. These examples depicted behavior differences between low and high scorers, incorporating micro-level behaviors like “smiles” and “makes responsive sounds” to combine the advantages of holistically processed behavioral information and the perception of specific behavioral acts (Funder et al., 2000). For the formulation of the rating items, we leaned on *The Münster Behavior Coding-System* (M-BeCoSy; Grünberg et al., 2018).

We divided task- and member-focus into three subdimensions that recurrently have been identified as core elements of the superordinate behavioral constructs (Bass, 1990; Burke et al., 2006; Yukl, 2008, 2012; Yukl et al., 2002, 2009). Task focus subdimensions were (a) “directs the group to its goals” (ICC [2, k] = .83), (b) “establishes structure” (ICC [2, k] = .78), and (c) “enforces efficiency” (ICC [2, k] = .91). Member-focus subdimensions were (a) “supports/acts considerately” (ICC [2, k] = .88), (b) “acknowledges/appreciates group members” (ICC [2, k] = .84), and (c) “empowers through collaboration” (ICC [2, k] = .91). Ratings for these subdimensions were ex-post aggregated to more global ratings of task-focus (α = .87) and member-focus (α = .93). Less is known about the internal dimensional structure of interpersonal calm behavior (Leising & Bleidorn, 2011). Thus, we rated it as a broader behavioral construct (ICC [2, k] = .63) defined as “the extent to which one controls one’s emotions, handles stress well, and reacts in a calm manner” (Breil et al., 2021; p. 229) comprising behaviors like “relaxed position,” “calm expression and gestures,” “does not break up sentences,” “no uncertain queries,” “no justification,” and “no oversensitive reactions” (Breil et al., 2021, 2022). See the Codebook at https://osf.io/6s9uf/ for a detailed breakdown of the specific behaviors that comprise each of the behavioral dimensions (Section 2.2 Behavioral Coding Instructions).

#### Interpersonal Impressions

After each round, group members reported their impressions on the respective group leader. Perceived assertiveness (“This person is assertive.”), trustworthiness (“This person is trustworthy.”) and calmness (“This person is calm.”) were rated on six-point scales ranging from 1 (*does not apply at all*) to 6 (*applies perfectly*). We computed target effects based on the social relations model (Back & Kenny, 2010) to capture individual differences in being seen as assertive, trustworthy, and calm. Target effects were computed in *R* using the *TripleR* package (Schönbrodt et al., 2012). Partner effect reliability was .68 for assertiveness, .47 for trustworthiness, and .49 for calmness. Partner effect reliabilities should not be compared against standards of conventional internal consistency coefficients—they are inherently lower, especially in small groups, but the variance components attributable to the target can be meaningfully associated with other variables (Bonito & Kenny, 2010).

#### Leadership Evaluations

Along with the interpersonal impression ratings, the group members provided leadership evaluations on the respective group leader after each round. Leadership emergence and effectiveness were measured with eight items each on six-point scales ranging from 1 (*does not apply at all*) to 6 (*applies perfectly*). Leadership emergence items covered appearing leaderlike (e.g., “I can well imagine this person as a leader.”), assuming responsibility (e.g., “This person takes on responsibility.”), and taking the leadership role (e.g., “This person assumes leadership duties in the group.”). Leadership effectiveness items captured directing the group toward achieving their goals (e.g., “This person fosters the achievement of group goals through their leadership behavior.”) and satisfaction with the leadership (e.g., “This person contributes to overall satisfaction through their leadership behavior.”) as the decisive aspects of leader performance as well as more direct assessments of this performance (e.g., “This person is an effective leader.”). We computed target effects for these evaluations. The partner effect reliabilities of the eight items ranged from .58 to .71 for leadership emergence and .52 to .65 for effectiveness. We then aggregated the target effects to form evaluations of leadership emergence (α = .96) and leadership effectiveness (α = .97).

### Analytical Approach

First, we calculated bivariate correlations between all variables to derive general associations between variables, initial support for the predicted pathways, and indications for cross-paths. We used group-mean-centered values of personality traits, behavioral ratings, and target effects of interpersonal impressions and leader evaluations to account for the hierarchical data structure (participants nested in groups). To test for differences between bivariate correlations, we computed Williams’ (1959) *t* (Hittner et al., 2003) and report Cohen’s (1988) *q* as effect size.

Subsequently, we tested the proposed pathways (see Figure 1) and hypotheses by computing a multiple mediator model (MMM; Preacher & Hayes, 2008) based on the group-mean-centered data while also considering cross-paths indicated in the correlation analysis. Given that leadership emergence and effectiveness processes primarily concern differences within, rather than between, groups (Judge et al., 2002), using group-mean-centered data is a standard and efficient approach to account for between-group variance, like experimental conditions, variations in group size, or gender distribution differences.

We conducted data preparation, descriptive analysis, and correlation analysis in R, whereas we used *Mplus* to specify the MMM. We used a nonparametric bootstrapping approach implemented in Mplus to compute 95% confidence intervals (CIs) for the indirect effects to check whether the 95% CIs of the behavioral pathways linking personality with leadership outcomes preclude zero. To test for differences between indirect effects and specific path coefficients, we also used bootstrapping to check whether the corresponding 95% CIs of these differences preclude zero. The number of bootstrap samples was 10,000. We conservatively computed two-sided *p* values/95%-confidence intervals in all instances.

## Results

### Descriptive Statistics and Bivariate Correlations

Table 1 provides descriptive statistics and intercorrelations of all measures used in our analysis. The bivariate correlations provided initial insights into the proposed pathways between personality traits and evaluated leadership outcomes. For the extraversion pathway, all component variables (i.e., extraversion, rated task-focused behavior, and being seen as assertive) were positively correlated with each other and with both evaluations of leadership emergence and effectiveness. Hence, the bivariate correlations support the expected effect of the extraversion pathway as well as the individual connections between its component variables as proposed in Hypothesis 1a. For the agreeableness pathway, the correlations between all component variables (i.e., agreeableness, rated member-focused behavior, and being seen as trustworthy) were positive. Being seen as trustworthy was positively correlated with evaluations of leadership emergence and effectiveness, which provides initial evidence in favor of Hypothesis 1b. For the emotional stability pathway, whereas not all component variables (i.e., emotional stability, rated calm behavior, and being seen as calm) were significantly correlated with each other, the postulated connections as proposed in the pathway all showed significant correlations. Namely, emotional stability was correlated with rated calm behavior, which was correlated with being seen as calm. Furthermore, regarding the final link to evaluated leadership outcomes, being seen as calm was positively correlated with evaluations of leadership effectiveness. Taken together, these correlations provide initial support for the pathway from emotional stability to evaluated leadership outcomes as proposed in Hypothesis 1c.

**Table 1.** Descriptive Statistics and Bivariate Correlations.

Model variables	*n* ^a^	*M*	*SD*	1	2	3	4	5	6	7	8	9	10	11	12	13	14
1. Extraversion	364	3.38	0.64	-	.05	**.35**	**.22**	−.02	−.02	**.20**	−.02	.00	**.23**	**.21**	.02	−.01	.03
2. Agreeableness	364	3.81	0.53		-	**.27**	−.04	**.15**	.04	−.05	**.11**	.00	.01	.04	.00	**−.15**	.02
3. Emotional stability	364	3.28	0.69			-	**.15**	−.05	**.12**	**.13**	−.05	−.02	**.16**	**.12**	.01	**.17**	**.11**
4. Task-focused behavior	363	3.46	1.09				-	−.01	**.14**	**.55**	**.15**	**−.15**	**.58**	**.51**	**.31**	**.16**	.09
5. Member-focused behavior	363	3.57	1.20					-	**.46**	−.04	**.36**	**.26**	.09	**.26**	−.05	−.10	.01
6. Calm behavior	363	3.61	0.94						-	.04	**.18**	**.23**	.07	**.19**	**.14**	**.15**	−.03
7. Being seen as assertive	360	4.10	0.80							-	**.31**	−.01	**.87**	**.70**	.04	**.17**	**.13**
8. Being seen as trustworthy	360	4.48	0.68								-	**.49**	**.42**	**.62**	.02	−.02	−.01
9. Being seen as calm	360	4.57	0.69									-	.08	**.31**	**−.17**	**.12**	−.06
10. Leadership emergence	360	4.02	0.71										-	**.86**	.05	**.15**	**.13**
11. Leadership effectiveness	360	4.06	0.74											-	.03	**.14**	.06
Controls																	
12. Round (1-5)	364	2.85	1.35												-	.03	.03
13. Gender (0/1 = women/men)	364	.40	.49													-	.01
14. Group performance^b^	362	58.1	15.1														-

*Note.* Means and standard deviations were calculated on raw scores. Correlations were calculated on group-mean-centered scores to account for nesting in groups. Correlations printed in bold were significant at the *p* < .05 level.

a Few participants dropped out due to technical difficulties during the online Zoom meeting resulting in some missing observations of behaviors, interpersonal impressions, leadership evaluations and group performance scores. ^b^Higher values on this variable indicate larger deviations of group rankings from expert rankings and thus lower performance.

To gain initial insights into the differential importance of the behavioral-perceptual pathways linking personality to evaluated leadership outcomes, we compared the correlations of the interpersonal impressions with evaluations of leadership emergence versus effectiveness. Being seen as assertive was more positively correlated with evaluations of leadership emergence than of effectiveness, Δ*r* = 0.17, *t*(357) = 12.41, *p* < .001, *q* = 0.47, which provides initial evidence in favor of Hypothesis 2a. Being seen as trustworthy, Δ*r* = 0.20, *t*(357) = 9.44, *p* < .001, *q* = 0.28, and being seen as calm, Δ*r* = 0.23, *t*(357) = 9.32, *p* < .001, *q* = 0.24, were more positively correlated with evaluations of leadership effectiveness than of emergence, which provides initial evidence in favor of Hypothesis 2b and 2c.

Finally, the bivariate correlations provided indications for cross paths between the three pathways that should be considered in the MMM. We added cross-paths to the MMM between emotional stability and rated task-focused behavior, between rated task-focused behavior and being seen as trustworthy/being seen as calm, between rated member-focused behavior and being seen as calm, and between rated calm behavior and being seen as trustworthy.

### Model Results

**Figure 2.**
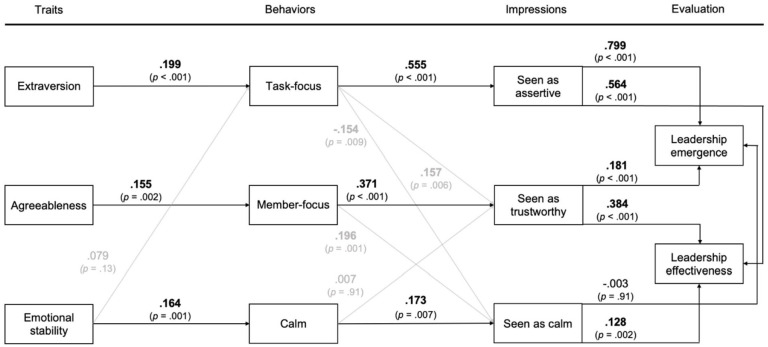
Model Results of the Behavioral Pathways Linking Personality to Leadership Outcomes. *Note.* Personality traits, expressed behaviors, interpersonal impressions, and evaluations were allowed to covary with each other (paths are not displayed for the sake of clarity). Results are presented as standardized path coefficients. Standardized path coefficients in bold were significant at the p < .05 level.

Figure 2 presents the results of the MMM including the three postulated pathways linking personality traits with evaluated leadership outcomes as well as the cross-paths identified in the correlation analysis. The model-fit indices, χ²(22) = 86.3, *p* < .001; root mean square error of approximation = .090; standardized root mean square residual = .035; comparative fit index = .963; Tucker–Lewis index = .913, suggested an adequate representation of the data (Bentler, 1990).

As theorized, the effect of interpersonal personality traits on evaluations of leadership emergence and effectiveness was mediated by the postulated interpersonal behaviors and impressions. In line with Hypothesis 1a, extraversion was expressed in rated task-focused behavior (β = .199; *p* < .001), which was reflected in being seen as assertive (β = .555, *p* < .001), which, in turn, was related to perceived leadership emergence (β = .799, *p* < .001) and effectiveness (β = .564, *p* < .001). In line with Hypothesis 1b, agreeableness was expressed in rated member-focused behavior (β = .155, *p* = .002), which was associated with being seen as trustworthy (β = .371, *p* < .001), which then was related to perceived leadership emergence (β = .181, *p* < .001) and effectiveness (β = .384, *p* < .001). Finally, in line with Hypothesis 1c, emotional stability was expressed in rated calm behavior (β = .164, *p* = .001) leading to impressions of being seen as calm (β = .173, *p* = .007). Being seen as calm was not significantly linked with perceived leadership emergence (β = −.003, *p* = .91), but was positively related to perceived leadership effectiveness (β = .128, *p* = .002).

Table 2 provides the direct effects and indirect effects for all three pathways, as well as a comparison between the indirect effects on evaluations of leadership emergence versus effectiveness for each pathway. As expected, the extraversion pathway exerted a positive indirect effect on both evaluations of leadership emergence (β = .088, 95% CI [.041, .142]) and effectiveness (β = .062, 95% CI [.029, .102]) supporting Hypothesis 1a. Likewise, the agreeableness pathway had positive and significant indirect effects on both evaluations of leadership emergence (β = .010, 95% CI [.003, .021]) and leadership effectiveness (β = .022, 95% CI [.007, .042]) supporting Hypothesis 1b. The emotional stability pathway, however, did not exert a significant indirect effect on perceived leadership emergence (β = .000, 95% CI [−.002, .002]), but only on perceived leadership effectiveness (β = .004, 95% CI [.000 .009]). Nevertheless, the indirect effect of emotional stability on perceived leadership effectiveness provides support for Hypothesis 1c.

**Table 2.** Direct and Indirect Effects of Personality Traits on Leadership Outcomes.

Personality traits	Leadership emergence					Leadership effectiveness					∆IE		
	DE	*p*_DE_	IE	95% CI		DE	*p*_DE_	IE	95% CI		IE	95% CI	
				LL_IE_	UL_IE_				LL_IE_	UL_IE_		LL_IE_	UL_IE_
Extraversion	**.055**	.030	**.088**	.041	.142	**.091**	.004	**.062**	.029	.102	**−.026**	−.044	−.011
Agreeableness	.014	.61	**.010**	.003	.021	.009	.79	**.022**	.007	.042	**.012**	.003	.022
Emotional stability	.041	.14	.000	−.002	.002	.036	.30	**.004**	.000	.009	**.004**	.001	.009

*Note.* DE = direct effect, IE = indirect effect, CI = confidence interval, LL = lower limit, UL = upper limit. DEs and IEs printed in bold are significant at the *p* < .05 level.

Regarding Hypotheses 2a-c, the comparison of indirect effects revealed that the effects of all three pathways differed significantly between the two evaluated leadership outcomes. In addition, we also compared the final paths between interpersonal impressions and evaluated leadership outcomes as the decisive link for these divergent effects. The results for the extraversion pathway support Hypothesis 2a. The indirect effect of extraversion on perceived leadership emergence was stronger than the indirect effect on perceived leadership effectiveness (Δβ = .026, 95% CI [.044, .011]) because being seen as assertive had a stronger impact on perceived leadership emergence than on perceived leadership effectiveness (Δβ = .236, 95% CI [.284, .184]). The other way around, and in line with Hypothesis 2b, the agreeableness pathway had a stronger indirect effect on perceived leadership effectiveness compared to perceived leadership emergence (Δβ = .012, 95% CI [.003, .022]) because being seen as trustworthy had a stronger impact on perceived leadership effectiveness than on perceived leadership emergence (Δβ = .203, 95% CI [.152, .255]). Finally, the emotional stability pathway provided evidence in favor of Hypothesis 2c. Emotional stability showed a stronger indirect effect on perceived leadership effectiveness compared to perceived leadership emergence (Δβ = .004, 95% CI [.001, .009]) because being seen as calm had a stronger impact on perceived leadership effectiveness than on perceived leadership emergence (Δβ = .131, 95% CI [.085, .180]).

### Robustness Tests

We provide supplemental results (see https://osf.io/6s9uf/) to the MMM reported in the main paper with control variables, that is, adding pathways to all behaviors, impressions, and evaluations for (a) the round in which the leader led the group (1-5), and (b) leader gender (0/1 = female/male). We also added pathways to all impressions and evaluations for (c) the objective group performance, that is, the total deviation of the submitted group rankings from expert rankings. It was computed for each participant based on the round in which they led the group by calculating the sum of the differences between the ranking positions of the 15 survival items in the submitted group ranking and the ranking positions in the correct ranking defined by experts for the respective group task (“Exploration,” 2006; Hall & Watson, 1970; Lafferty & Eady, 1973; Lafferty et al., 1974; Nemiroff & Pasmore, 2008). Note that higher values on the variable indicate larger deviations from the expert rankings and thus lower performance.

Controlling for rounds allowed us to examine the robustness of our findings against any systematic alterations that may have occurred across rounds. For instance, leaders may have learned from observing other participants, become more familiar with the task, felt less nervous due to increased familiarity with the other participants/overall setting, and become more aware of the evaluations made by others. Also, group members may have compared the current target leader to previous leaders. Controlling for gender is important as gender has been demonstrated to strongly affect how leader behaviors are evaluated (e.g., Eagly et al., 1992). Controlling for group performance allowed us to account for the possibility that leaders in groups performing objectively well may enjoy more favorable impression perceptions and leadership evaluations compared with leaders in groups with poorer performance.

We focus on the models without controls because they are the less complex and easier-to-interpret analysis. The models in the supplement show the same pattern of results and do not alter the main conclusions, that is, neither controlling for round, nor for gender, nor for group performance did substantially affect how personality was expressed in behaviors, how these behaviors were used to form impressions, and how these impressions were evaluated regarding leadership outcomes. As such, all path coefficients and (differences between) indirect effects that were significant in the original model reported in the main manuscript remained significant in the models controlling for round, gender, and group performance, respectively.

## Discussion

With this study, we follow calls (Antonakis et al., 2012; DeRue et al., 2011; Judge et al., 2002; Zaccaro et al., 2018) to examine leadership process models to open the “black box” (i.e., the unknown proximal mechanisms) explaining the enigmatic links between distal personality traits and leadership evaluations in groups. Specifically, we address calls (e.g., Banks et al., 2021; Blake et al., 2022; Fischer et al., 2023; Hu et al., 2019; Judge et al., 2004, 2009) to focus on truly behavioral constructs, which represent the key linking mechanism between personality traits and social outcomes in process models of personality (e.g., Back, 2021; Back et al., 2023). For this purpose, we drew on behavioral personality science and focused on three conceptually distinct interpersonal behaviors (rated task-focus, member-focus, and calmness) that represent the fundamental behavioral building blocks across a wide range of social situations including organizational, team, and leadership contexts (Breil et al., 2021, 2022; Leising & Bleidorn, 2011). These interpersonal behaviors reflect interpersonal big five personality traits (extraversion, agreeableness, and emotional stability) and evoke leadership-relevant interpersonal impressions (assertive, trustworthy, and calm) allowing us to unravel the why and how of the puzzling personality-leadership links in groups.

Utilizing multimethodological data from a large online group interaction study, we first showed that interpersonal personality traits affect evaluations of leadership outcomes indirectly via expressed interpersonal behaviors and impressions. We then used the behavioral pathways to unravel personality traits’ divergent main effects on leadership outcomes. We found extraversion to affect leadership outcomes via rated task-focused behavior and impressions of assertiveness (Hypothesis 1a), which were more strongly valued for evaluations of leadership emergence (Hypothesis 2a). In contrast, agreeableness/emotional stability affected leadership outcomes via rated member-focused/calm behavior and impressions of trustworthiness/calmness (Hypothesis 1b/c), which were more strongly valued for evaluations of leadership effectiveness (Hypothesis 2b/c).

### Unveiling Personality-Leadership Links Through Behavioral Pathways

This study underscores the value of adopting a behavioral pathway approach rooted in process models of personality (e.g., Back, 2021; Back et al., 2023) and core dimensions of interpersonal behavior informed by behavioral personality science (Breil et al., 2021, 2022; Leising & Bleidorn, 2011). Specifically, grounded in the agency/communion framework (e.g., Wojciszke et al., 2009) of interpersonal theory (e.g., Bakan, 1966; Dawood et al., 2018), we confirm the anticipated connections proposed between interpersonal personality traits (extraversion and agreeableness), interpersonal behaviors (rated task-focus and member-focus), and interpersonal impressions (assertiveness and trustworthiness). Yet, we not only add support to the foundational links posited in interpersonal theory but also showcase the potential to construct comprehensive behavioral-perceptual pathways based on these links. These personality-evoked behavioral pathways contribute to our understanding of the processes by which personality translates into interpersonal consequences, as demonstrated by group leadership dynamics.

Furthermore, we support initial findings uncovering a third distinct dimension of reliably observable interpersonal behavior, that is, interpersonal calm behavior (Leising & Bleidorn, 2011). We complement these findings by demonstrating that individual trait differences in emotional stability are expressed in rated interpersonal calm behavior, similar to extraversion/agreeableness triggering rated task-/member-focused behavior. Interindividual differences in rated interpersonal calm behavior were observable by interaction partners, translating to interaction partners’ impressions of calmness. The resulting behavioral pathway was found to be relevant for explaining social outcomes in business-relevant contexts (see Breil et al., 2021, 2022): Consistent with the main effects of emotional stability on leadership outcomes (Badura et al., 2022; DeRue et al., 2011; Ensari et al., 2011), albeit not being important for evaluations of rising as a leader, the calmness behavioral pathway had a positive impact on evaluations of leading effectively. In line with previous findings (Leising & Bleidorn, 2011), the effect of the calmness pathway was weaker compared with the other interpersonal domain pathways but still meaningful.

These findings add a more detailed understanding revolving around interpersonal calm behavior to a burgeoning body of research highlighting the benefits of individual resilience (e.g., Bowman, 2022; Hartmann et al., 2020; King et al., 2016), emotional stability (e.g., Ishaq et al., 2021; Li et al., 2012; Ormiston et al., 2022) and calmness (e.g., Klus & Müller, 2021) in workplace contexts. Specifically, we illuminate the calmness-related traits, behaviors, and impressions that play a role in the interpersonal domain of leadership processes, establishing ties between leaders and their followers (see also Razinskas & Hartmann, 2023). Yet, we emphasize that the findings of the interpersonal calmness pathway should be interpreted with somewhat more caution due to its novelty and limited establishment in the leadership literature as well as the overall weaker effects. Also, following trait activation theory (Tett & Guterman, 2000), it is likely that the importance of the interpersonal calmness pathway depends on the presence of situational triggers (see also Hirschmüller et al., 2015), such as time pressure (e.g., completing tasks within a narrow time frame), social pressure (e.g., being evaluated by others), and performance pressure (e.g., bonuses tied to group performance; Breil et al., 2021). Although it can be argued that such factors are prevalent in most real-world leadership scenarios, where important decisions, taking responsibility for group members’ needs, and achieving group goals often coincide with tight work-related deadlines, future research may pay closer attention to these boundary factors. This can contribute to a more nuanced understanding of the generalizability of the interpersonal calmness pathway across a broad spectrum of interpersonal situations.

### Understanding Distinct Effects of Personality on Evaluations of Leadership Emergence and Effectiveness

The key links of the causal mediation chain to explain personality trait’s divergent relation with leadership outcomes are the personality-evoked interpersonal impressions that were differently weighted by interaction partners in terms of evaluations of leadership emergence and effectiveness: Extraversion-evoked impressions of assertiveness were more valued in terms of leadership emergence, whereas agreeableness/emotional stability-evoked impressions of trustworthiness/calmness were more valued in terms of leadership effectiveness. These findings can be laid out in the agency/communion-framework of interpersonal theory (e.g., Bakan, 1966; Dawood et al., 2018; Wojciszke et al., 2009). For evaluations of leadership emergence, the agency component may be more influential because it pertains to the pursuit of individual goals such as self-oriented status and leadership attainment. Thus, the core agentic impression of assertiveness reflecting extraversion may be given greater weight. In contrast, for evaluations of leadership effectiveness, the communal component may become salient, when other-oriented action, such as managing group members’ needs and aligning group processes to commonly shared goals and satisfaction, gains importance. Hence, the core communal impression of trustworthiness reflecting agreeableness may be deemed more influential.

This aligns well with prototypical leadership theories (e.g., Epitropaki & Martin, 2004; Lord et al., 1984; Offermann et al., 1994), encompassing people’s ideas of prototypical leader characteristics that are conducive to rising to a leadership position. When visualizing a leader, followers may intuitively think of someone who is assertive rather than trustworthy/calm (e.g., Reichard et al., 2011). Thus, being perceived as assertive may be more valued in terms of evaluations of leadership emergence, whereas being seen as trustworthy/calm rather gains appreciation in terms of evaluations of leadership effectiveness. Indeed, Epitropaki and Martin (2004) found agentic leader attributes like “hard-working,” “strong,” and “energetic” among the most prototypical leader characteristics, whereas communal attributes like “sensitive,” “warm,” and “sympathetic” tended to be evaluated somewhat less characteristic. Similarly, Offermann et al. (1994) found dominant-assertive attributes to be more pronounced in prototypical leaders, whereas sensitive-trustworthy attributes were slightly more pronounced in prototypical *effective* leaders. Calmness characteristics are seldom included in prototypical leadership theories, which aligns with our finding that the calmness pathway is not relevant for leadership emergence.

### Limitations and Future Research

This study was conducted in a laboratory setting using a student sample. Although participants were mostly business students and received financial incentives, external validity needs to be tested in more natural (business) environments. In a similar vein, we examined leadership evaluations within a brief time frame. We found clear distinctions in perceptions of leadership emergence versus effectiveness and, most importantly, personality traits showed distinct effects with personality-evoked impressions being differently evaluated in terms of the two leadership outcomes. However, questions about becoming a leader and leader effectiveness typically become urgent at different timely stages with leadership emergence processes being more important at the beginning and the focus shifting to leadership effectiveness as time progresses (Ong et al., 2016). Future research should replicate our behavioral pathways over extended time frames (e.g., multi-methodological laboratory studies that videotape group interactions at multiple measurement points over several weeks; Leckelt et al., 2015). Also, future research may complement continuous assessments in real-life teams with higher stakes (Wrzus & Mehl, 2015) using ambulatory assessment methods such as daily-diary methods or interaction-based experience sampling (e.g., Harari et al., 2016; Larson & Csikszentmihalyi, 2014).

Also, while a pattern of divergent main effects between personality traits and leadership outcomes emerged in the literature, there is substantial between-study variation and some contradictory findings to this pattern (e.g., Badura et al., 2022; Blake et al., 2022; Ensari et al., 2011; Judge et al., 2002), suggesting the presence of moderators. We add to the understanding of how personality relates to evaluated leadership outcomes, facilitating to identify such moderators (Fischer et al., 2017): Moderators can intervene at any stage of the personality-leadership chain and strengthen or weaken specific links (e.g., Grosz et al., 2020). For example, our research was conducted in virtual groups due to COVID-19-restrictions, which may have influenced the expression of personality in behaviors. For instance, following trait-activation theory (Tett & Guterman, 2000), the expression of low emotional stability in nervous interpersonal behaviors may be reduced when there is less confrontative face-to-face interaction (less trait-relevant situation; e.g., Hirschmüller et al., 2015). In terms of the link between behaviors and impressions, some information may be lost due to limitations in video quality. Also, the evaluation of impressions may have been impacted—trustworthiness may be more valued in virtual groups (Breuer et al., 2016). Future research may replicate our findings in face-to-face groups and move on to other contextual moderators. According to the path-goal theory of leadership (House, 1971), the effectiveness of leadership behaviors is contingent upon followers’ specific needs (e.g., task clarity and social support) and broader situational factors (e.g., urgent workload and hierarchical structures), which may thus offer a promising theoretical avenue to identify boundary conditions rendering personality-evoked leadership behaviors more or less effective. This will add a more nuanced understanding of the “when” to the “how/why” of personality-leadership links.

Finally, we ultimately cannot claim to demonstrate causality as deriving clear causal inferences in mediation models is challenging. The model in the present study was conceptually based on the directional logic of process models of personality (e.g., Back, 2021; Back et al., 2023) and leadership process models (e.g., Antonakis et al., 2012; Zaccaro et al., 2018), positing a causal chain: traits influence behaviors, which shape interpersonal impressions, ultimately impacting leadership evaluations. Methodologically, this study established temporal precedence through multiple measurement time points and employed multiple data sources. As such, expressed behaviors could not impact previously assessed personality traits, just as the group members’ mutual impressions could not impact external raters’ coded behaviors. However, drawing clear causal inferences remains difficult due to multiple plausible models that may fit the data (e.g., mediation vs. confounding/suppression effects; MacKinnon et al., 2000). Particularly, omitted confounding variables, influencing both mediators and dependent variables, can distort indirect effect estimates. For example, a leader’s rated task-focused behavior may have been influenced by their technical setup (ensuring better or worse visibility/audibility), which in turn may have also influenced leadership evaluations. This is a common problem in mediation analysis, and it is impossible to control for all potentially influential variables. Yet, follow-up randomized experimental studies could directly manipulate mediators to minimize their correlation with other variables (Bullock et al., 2010).

## Conclusion

Adopting a comparative behavioral pathway approach enabled us to reveal important differences and similarities in the behavioral-perceptual processes that make a person emerge as a group leader versus those that make an effective group leader. Thereby, we contribute to the nascent literature on leadership process models by marrying it with the literature streams of process models of personality and behavioral personality science, creating a powerful framework to unravel personality’s enigmatic leadership effects. Adopting such a behavioral-perceptual pathway approach is not restricted to leadership contexts but opens a promising avenue to illuminate personality effects on a broad range of social outcomes such as status, popularity, and interpersonal attraction.

## Supplemental Material

sj-docx-1-psp-10.1177_01461672241246388 – Supplemental material for Differential Behavioral Pathways Linking Personality to Leadership Emergence and Effectiveness in Groups Supplemental material, sj-docx-1-psp-10.1177_01461672241246388 for Differential Behavioral Pathways Linking Personality to Leadership Emergence and Effectiveness in Groups by Tobias M. Härtel, Felix Hoch and Mitja D. Back in Personality and Social Psychology Bulletin

## Notes

1. The empirical relationships found between the two remaining, less interpersonal big five traits, conscientiousness and openness, with the three basic types of interpersonal behavior support the conceptual choice to focus on the three interpersonal big five traits. As such, conscientiousness was not significantly associated with any of the interpersonal behaviors, *r*_Conscientiousness, task-focus_ = -.04, *t*(361) = -0.72, *p* = .47; *r*_Conscientiousness, member-focus_ = .03, *t*(361) = 0.58, *p* = .57, *r*_Conscientiousness, calm behavior_ = .08, *t*(361) = 1.53, *p* = .13. Openness was not expressed in member-focused behavior, *r* = .06, *t*(361) = 1.15, *p* = .25, and calm behavior, *r* = .07, *t*(361) = 1.41, *p* = .16, but it showed a small, albeit significant, association with task-focused behavior, *r* = .10, *t*(361) = 2.01, *p* = .046. Yet, the postulated effect of extraversion on task-focused behavior, *r* = .22, *t*(361) = 4.20, *p* < .001, was significantly stronger than the effect of openness on task-focused behavior, Δ*r* = 0.12, *t*(360) = 1.93, *p* = .050, *q* = 0.12, with the effect of extraversion being more than twice as potent.

2. The instructions differed slightly between two sets of conditions, namely “competitive” versus “noncompetitive” and “authoritarian” versus “participative.” In the competitive condition, the variable payoff depended on the group’s performance relative to the other same session group, whereas the payoff in the noncompetitive condition depended solely on the group’s own performance. In the authoritarian condition, leaders were instructed to utilize an authoritarian directive leadership style and to establish a steep hierarchy, whereas leaders in the participative condition were instructed to utilize a participative inclusive leadership style and to establish a flat hierarchy. The experimental conditions were introduced to investigate unrelated research questions in other projects. We conducted our analyses based on group-mean-centered data (see section “Analytical Approach”), as only the within-group effects are of interest for this study. In doing so, we aimed to minimize the impact of the experimental conditions on the present findings and conclusions. Also, the experimental conditions were not linked to differences in the group performance in terms of task solution quality, that is, the total deviation between the submitted group rankings of the items for survival and expert rankings. To test this, we computed a multiple linear regression predicting group performance with two dummy variables representing the two sets of experimental conditions (noncompetitive/competitive = 0/1, participative/authoritarian = 0/1) and their interaction effect. The experimental conditions did not explain significant variance in group performance, *R*^2^ = .01, *R*^2^_Adj._ = .01, *F*(3, 358) = 1.72, *p* = .16, and neither the main effects of the two sets of experimental conditions, β_Competitive_ = -1.18, *t*(358) = -0.53, *p* = .60; β_Authoritarian_ = -1.74, *t*(358) = 0.77, *p* = .44, nor their interaction effect, β_Competitive x authoritarian_ = 5.89, *t*(358) = 1.85, *p* = .065, predicted group performance. Yet, even though we used group-mean-centered data to mitigate between-group influences and there were no between-group differences in terms of group performance introduced by the experimental conditions, the generalizability may still be somewhat limited by persistent unknown influences of the experimental conditions.

3. Whereas leadership emergence is often investigated in leaderless groups, it also occurs and is researched in contexts with formally assigned leader roles (e.g., Badura et al., 2022). Assigning leader roles allows the simultaneous examination of perceptions of emergent and effective leadership, which was the purpose of this study. Also, assigned leader roles facilitate to focus all group members’ attention on the target leader. There was meaningful variation in how assigned leaders were perceived as emerging leaders (*SD* = 0.71), similar to the variation in the interpersonal impressions (see Table 1).

4. For two groups, one rater did not rate the respective group because of a personal connection to a participant. Hence, two groups (nine leaders) were rated by five raters.

5. Three recordings included only the audio track of the leader without visual material because two participants had technical issues with their webcam and one participant withdraw their consent to analyze the visual material.

## References

[bibr1-01461672241246388] Abele A. E. Cuddy A. J. C. Judd C. M. Yzerbyt V. Y. (2008). Fundamental dimensions of social judgment. European Journal of Social Psychology, 38(7), 1063–1065. 10.1002/ejsp.574

[bibr2-01461672241246388] Abele A. E. Wojciszke B. (2007). Agency and communion from the perspective of self versus others. Journal of Personality and Social Psychology, 93(5), 751–763. 10.1037/0022-3514.93.5.751 17983298

[bibr3-01461672241246388] Ames D. R. Flynn F. J. (2007). What breaks a leader: The curvilinear relation between assertiveness and leadership. Journal of Personality and Social Psychology, 92(2), 307–324. 10.1037/0022-3514.92.2.307 17279851

[bibr4-01461672241246388] Anderson C. John O. P. Keltner D. Kring A. M. (2001). Who attains social status? Effects of personality and physical attractiveness in social groups. Journal of Personality and Social Psychology, 81(1), 116–132. 10.1037/0022-3514.81.1.116 11474718

[bibr5-01461672241246388] Antonakis J. Day D. V. Schyns B. (2012). Leadership and individual differences: At the cusp of a renaissance. The Leadership Quarterly, 23(4), 643–650. 10.1016/j.leaqua.2012.05.002

[bibr6-01461672241246388] Back M. D. (2021). Social interaction processes and personality. In Rauthmann J. (Ed.), The handbook of personality dynamics and processes (pp. 183–226). Elsevier. 10.1016/C2017-0-00935-7

[bibr7-01461672241246388] Back M. D. Baumert A. Denissen J. J. A. Hartung F.-M. Penke L. Schmukle S. C. Schönbrodt F. D. Schröder-Abé M. Vollmann M. Wagner J. Wrzus C. (2011). PERSOC: A unified framework for understanding the dynamic interplay of personality and social relationships. European Journal of Personality, 25(2), 90–107. 10.1002/per.811

[bibr8-01461672241246388] Back M. D. Branje S. Eastwick P. W. Human L. J. Penke L. Sadikaj G. Slatcher R. B. Thielmann I. van Zalk M. H. W. Wrzus C. (2023). Personality and social relationships: What do we know and where do we go. Personality Science, 4, 1–32. 10.5964/ps.7505

[bibr9-01461672241246388] Back M. D. Kenny D. A. (2010). The social relations model: How to understand dyadic processes. Social and Personality Psychology Compass, 4(10), 855–870. 10.1111/j.1751-9004.2010.00303.x

[bibr10-01461672241246388] Badura K. L. Galvin B. M. Lee M. Y. (2022). Leadership emergence: An integrative review. Journal of Applied Psychology, 107(11), 2069–2100. 10.1037/apl0000997 34968077

[bibr11-01461672241246388] Bakan D. (1966). The duality of human existence: An essay on psychology and religion. Rand McNally. 10.1002/1520-6696(196801)4:1<88::AID-JHBS2300040115>3.0.CO;2-E

[bibr12-01461672241246388] Banks G. C. Woznyj H. M. Mansfield C. A. (2021). Where is “behavior” in organizational behavior? A call for a revolution in leadership research and beyond. The Leadership Quarterly, 34, 101581. 10.1016/j.leaqua.2021.101581

[bibr13-01461672241246388] Barford K. A. Zhao K. Smillie L. D. (2015). Mapping the interpersonal domain: Translating between the big five, HEXACO, and interpersonal circumplex. Personality and Individual Differences, 86, 232–237. 10.1016/j.paid.2015.05.038

[bibr14-01461672241246388] Bass B. M. (1990). Bass & Stogdill’s handbook of leadership: Theory, research, and managerial applications (3rd ed.). The Free Press.

[bibr15-01461672241246388] Bentler P. M. (1990). Comparative fit indexes in structural models. Psychological Bulletin, 107(2), 238–246. 10.1037/0033-2909.107.2.238 2320703

[bibr16-01461672241246388] Blake A. B. Luu V. H. Petrenko O. V. Gardner W. L. Moergen K. J. N. Ezerins M. E. (2022). Let’s agree about nice leaders: A literature review and meta-analysis of agreeableness and its relationship with leadership outcomes. The Leadership Quarterly, 33(1), 101593. 10.1016/J.LEAQUA.2021.101593

[bibr17-01461672241246388] Bonito J. A. Kenny D. A. (2010). The measurement of reliability of social relations components from round-robin designs. Personal Relationships, 17(2), 235–251. 10.1111/j.1475-6811.2010.01274.x

[bibr18-01461672241246388] Borkenau P. Mauer N. Riemann R. Spinath F. M. Angleitner A. (2004). Thin slices of behavior as cues of personality and intelligence. Journal of Personality and Social Psychology, 86(4), 599–614. 10.1037/0022-3514.86.4.599 15053708

[bibr19-01461672241246388] Bottger P. C. (1984). Expertise and air time as bases of actual and perceived influence in problem-solving groups. Journal of Applied Psychology, 69(2), 214–221. 10.1037/0021-9010.69.2.214

[bibr20-01461672241246388] Bowman A. (2022). Leadership and resilience: Where the literature stands. Journal of Leadership Studies, 16(2), 33–41. 10.1002/jls.21815

[bibr21-01461672241246388] Breil S. M. Forthmann B. Back M. D. (2021). Measuring distinct social skills via multiple speed assessments. European Journal of Psychological Assessment, 38, 163–238. 10.1027/1015-5759/a000657

[bibr22-01461672241246388] Breil S. M. Lievens F. Forthmann B. Back M. D. (2022). Interpersonal behavior in assessment center role-play exercises: Investigating structure, consistency, and effectiveness. Personnel Psychology, 76, 759–795. 10.1111/peps.12507

[bibr23-01461672241246388] Breuer C. Hüffmeier J. Hertel G. (2016). Does trust matter more in virtual teams? A meta-analysis of trust and team effectiveness considering virtuality and documentation as moderators. Journal of Applied Psychology, 101(8), 1151–1177. 10.1037/apl0000113 27228105

[bibr24-01461672241246388] Bullock J. G. Green D. P. Ha S. E. (2010). Yes, but what’s the mechanism? (Don’t expect an easy answer). Journal of Personality and Social Psychology, 98(4), 550–558. 10.1037/a0018933 20307128

[bibr25-01461672241246388] Burke C. S. Stagl K. C. Klein C. Goodwin G. F. Salas E. Halpin S. M. (2006). What type of leadership behaviors are functional in teams? A meta-analysis. The Leadership Quarterly, 17(3), 288–307. 10.1016/j.leaqua.2006.02.007

[bibr26-01461672241246388] Cheng J. T. Tracy J. L. Foulsham T. Kingstone A. Henrich J. (2013). Two ways to the top: Evidence that dominance and prestige are distinct yet viable avenues to social rank and influence. Journal of Personality and Social Psychology, 104(1), 103–125. 10.1037/a0030398 23163747

[bibr27-01461672241246388] Cohen J. (1988). Statistical power analysis for the behavioral sciences (2nd ed.). Lawrence Erlbaum.

[bibr28-01461672241246388] Danner D. Rammstedt B. Bluemke M. Lechner C. Berres S. Knopf T. Soto C. J. John O. P. (2019). Das Big Five Inventar 2: Validierung eines Persönlichkeitsinventars zur Erfassung von 5 Persönlichkeitsdomänen und 15 Facetten [The German Big Five Inventory 2: Measuring Five Personality Domains and 15 Facets]. Diagnostica, 65(3), 121–132. 10.1026/0012-1924/a000218

[bibr29-01461672241246388] Dawood S. Dowgwillo E. A. Wu L. Z. Pincus A. L. (2018). Contemporary integrative interpersonal theory of personality. In Zeigler-Hill V. Shackelford T. K. (Eds.), The SAGE handbook of personality and individual differences: The science of personality and individual differences (pp. 171–200). Sage. 10.4135/9781526451163.n8

[bibr30-01461672241246388] DeRue D. S. Nahrgang J. D. Wellman N. Humphrey S. E. (2011). Trait and behavioral theories of leadership: An integration and meta-analytic test of their relative validity. Personnel Psychology, 64(1), 7–52. 10.1111/j.1744-6570.2010.01201.x

[bibr31-01461672241246388] Eagly A. H. Makhijani M. G. Klonsky B. G. (1992). Gender and the evaluation of leaders: A meta-analysis. Psychological Bulletin, 111(1), 3–22. https://doi.org/doi.org/10.1037/0033-2909.111.1.3

[bibr32-01461672241246388] Ennis G. Happell B. Reid-Searl K. (2015). Clinical leadership in mental health nursing: The importance of a calm and confident approach. Perspectives in Psychiatric Care, 51(1), 57–62. 10.1111/ppc.12070 24734981

[bibr33-01461672241246388] Ensari N. Riggio R. E. Christian J. Carslaw G. (2011). Who emerges as a leader? Meta-analyses of individual differences as predictors of leadership emergence. Personality and Individual Differences, 51(4), 532–536. 10.1016/j.paid.2011.05.017

[bibr34-01461672241246388] Epitropaki O. Martin R. (2004). Implicit leadership theories in applied settings: Factor structure, generalizability, and stability over time. Journal of Applied Psychology, 89(2), 293–310. 10.1037/0021-9010.89.2.293 15065976

[bibr35-01461672241246388] Exploration: Then and now—survival! Lesson. (2006, September 25). NASA. http://www.nasa.gov/stem-ed-resources/jamestown-survival.html

[bibr36-01461672241246388] Ferrin D. L. Dirks K. T. (2002). Trust in leadership: Meta-analytic findings and implications for research and practice. Journal of Applied Psychology, 87(4), 611–628. 10.1037/0021-9010.87.4.611 12184567

[bibr37-01461672241246388] Fischer T. Dietz J. Antonakis J. (2017). Leadership process models: A review and synthesis. Journal of Management, 43(6), 1726–1753. 10.1177/0149206316682830

[bibr38-01461672241246388] Fischer T. Hambrick D. C. Sajons G. B. Van Quaquebeke N. (2023). Leadership science beyond questionnaires. The Leadership Quarterly, 34(6), 101752. 10.1016/j.leaqua.2023.101752

[bibr39-01461672241246388] Funder D. C. Furr R. M. Colvin C. R. (2000). The Riverside Behavioral Q-sort: A tool for the description of social behavior. Journal of Personality, 68(3), 451–489. 10.1111/1467-6494.00103 10831309

[bibr40-01461672241246388] Grosz M. P. Leckelt M. Back M. D. (2020). Personality predictors of social status attainment. Current Opinion in Psychology, 33, 52–56. 10.1016/j.copsyc.2019.07.023 31400659

[bibr41-01461672241246388] Grünberg M. Mattern J. Geukes K. Küfner A. C. P. Back M. D. (2018). Assessing group interactions in personality psychology: The Münster Behavior Coding-System (M-BeCoSy). In Brauner E. Boos Kolbe M. (Eds.), Cambridge handbook of group interaction analysis (pp. 602–611). Cambridge University Press. 10.1017/9781316286302.042

[bibr42-01461672241246388] Hall J. Watson W. H. (1970). The effects of a normative intervention on group decision-making performance. Human Relations, 23(4), 299–317. 10.1177/001872677002300404

[bibr43-01461672241246388] Hanna A. A. Smith T. A. Kirkman B. L. Griffin R. W. (2021). The emergence of emergent leadership: A comprehensive framework and directions for future research. Journal of Management, 47(1), 76–104. 10.1177/0149206320965683

[bibr44-01461672241246388] Harari G. M. Lane N. D. Wang R. Crosier B. S. Campbell A. T. Gosling S. D. (2016). Using smartphones to collect behavioral data in psychological science: Opportunities, practical considerations, and challenges. Perspectives on Psychological Science, 11(6), 838–854. 10.1177/1745691616650285 27899727 PMC5572675

[bibr45-01461672241246388] Hare A. P. (1976). Handbook of small group research (2nd ed.). The Free Press.

[bibr46-01461672241246388] Hare A. P. (1981). Group size. American Behavioral Scientist, 24(5), 695–708. 10.1177/000276428102400507

[bibr47-01461672241246388] Härtel T. M. Leckelt M. Grosz M. P. Küfner A. C. P. Geukes K. Back M. D. (2021). Pathways from narcissism to leadership emergence in social groups. European Journal of Personality, 35(5), 1–23. 10.1177/08902070211046266

[bibr48-01461672241246388] Hartmann S. Weiss M. Newman A. Hoegl M. (2020). Resilience in the workplace: A multilevel review and synthesis. Applied Psychology, 69(3), 913–959. 10.1111/apps.12191

[bibr49-01461672241246388] Hirschmüller S. Egloff B. Schmukle S. C. Nestler S. Back M. D. (2015). Accurate judgments of neuroticism at zero acquaintance: A question of relevance. Journal of Personality, 83(2), 221–228. 10.1111/jopy.12097 24655148

[bibr50-01461672241246388] Hittner J. B. May K. Silver N. C. (2003). A Monte Carlo evaluation of tests for comparing dependent correlations. The Journal of General Psychology, 130(2), 149–168. 10.1080/00221300309601282 12773018

[bibr51-01461672241246388] Hoffman B. J. Woehr D. J. Maldagen-Youngjohn R. Lyons B. D. (2011). Great man or great myth? A quantitative review of the relationship between individual differences and leader effectiveness. Journal of Occupational and Organizational Psychology, 84(2), 347–381. 10.1348/096317909X485207

[bibr52-01461672241246388] Hogan J. Holland B. (2003). Using theory to evaluate personality and job-performance relations: A socioanalytic perspective. Journal of Applied Psychology, 88(1), 100–112. 10.1037/0021-9010.88.1.100 12675398

[bibr53-01461672241246388] Hogg M. A. (2001). A social identity theory of leadership. Personality and Social Psychology Review, 5(3), 184–200. 10.1207/S15327957PSPR0503_1

[bibr54-01461672241246388] Hopwood C. J. (2018). Interpersonal dynamics in personality and personality disorders. European Journal of Personality, 32(5), 499–524. 10.1002/per.2155

[bibr55-01461672241246388] House R. J. (1971). A path goal theory of leader effectiveness. Administrative Science Quarterly, 16(3), 321–339. 10.2307/2391905

[bibr56-01461672241246388] Hu J. Zhang Z. Jiang K. Chen W. (2019). Getting ahead, getting along, and getting prosocial: Examining extraversion facets, peer reactions, and leadership emergence. Journal of Applied Psychology, 104(11), 1369–1386. 10.1037/apl0000413 30998025

[bibr57-01461672241246388] Ishaq E. Bashir S. Khan A. K. (2021). Paradoxical leader behaviors: Leader personality and follower outcomes. Applied Psychology, 70(1), 342–357. 10.1111/apps.12233

[bibr58-01461672241246388] Judge T. A. Bono J. E. Ilies R. Gerhardt M. W. (2002). Personality and leadership: A qualitative and quantitative review. Journal of Applied Psychology, 87(4), 765–780. 10.1037/0021-9010.87.4.765 12184579

[bibr59-01461672241246388] Judge T. A. Piccolo R. F. Ilies R. (2004). The forgotten ones? The validity of consideration and initiating structure in leadership research. Journal of Applied Psychology, 89(1), 36–51. https://doi.org/https://doi.org/10.1037/0021-9010.89.1.36 14769119 10.1037/0021-9010.89.1.36

[bibr60-01461672241246388] Judge T. A. Piccolo R. F. Kosalka T. (2009). The bright and dark sides of leader traits: A review and theoretical extension of the leader trait paradigm. The Leadership Quarterly, 20(6), 855–875. 10.1016/j.leaqua.2009.09.004

[bibr61-01461672241246388] King D. D. Newman A. Luthans F. (2016). Not if, but when we need resilience in the workplace. Journal of Organizational Behavior, 37(5), 782–786. 10.1002/job.2063

[bibr62-01461672241246388] Klus M. F. Müller J. (2021). The digital leader: What one needs to master today’s organisational challenges. Journal of Business Economics, 91(8), 1189–1223. 10.1007/s11573-021-01040-1

[bibr63-01461672241246388] Küfner A. C. P. Nestler S. Back M. D. (2013). The two pathways to being an (un-)popular narcissist. Journal of Personality, 81(2), 184–195. 10.1111/j.1467-6494.2012.00795.x 22583074

[bibr64-01461672241246388] Lafferty J. C. Eady P. M. (1973). The subarctic survival situation. Experiential Learning Methods.

[bibr65-01461672241246388] Lafferty J. C. Eady P. M. Pond A. (1974). The desert survival situation (8th ed.). Experiential Learning Methods.

[bibr66-01461672241246388] Larson R. Csikszentmihalyi M. (2014). The experience sampling method. In Csikszentmihalyi M. (Ed.), Flow and the foundations of positive psychology: The collected works of Mihaly Csikszentmihalyi (pp. 21–34). Springer. 10.1007/978-94-017-9088-8_2

[bibr67-01461672241246388] Leckelt M. Küfner A. C. P. Nestler S. Back M. D. (2015). Behavioral processes underlying the decline of narcissists’ popularity over time. Journal of Personality and Social Psychology, 109(5), 856–871. 10.1037/pspp0000057 26191958

[bibr68-01461672241246388] Legood A. van der Werff L. Lee A. Den Hartog D. (2021). A meta-analysis of the role of trust in the leadership-performance relationship. European Journal of Work and Organizational Psychology, 30(1), 1–22. 10.1080/1359432X.2020.1819241

[bibr69-01461672241246388] Leising D. Bleidorn W. (2011). Which are the basic meaning dimensions of observable interpersonal behavior? Personality and Individual Differences, 51(8), 986–990. 10.1016/j.paid.2011.08.003

[bibr70-01461672241246388] Li Y. Chun H. Ashkanasy N. M. Ahlstrom D. (2012). A multi-level study of emergent group leadership: Effects of emotional stability and group conflict. Asia Pacific Journal of Management, 29(2), 351–366. 10.1007/s10490-012-9298-4

[bibr71-01461672241246388] Lord R. G. De Vader C. L. Alliger G. M. (1986). A meta-analysis of the relation between personality traits and leadership perceptions: An application of validity generalization procedures. Journal of Applied Psychology, 71(3), 402–410. 10.1037/0021-9010.71.3.402

[bibr72-01461672241246388] Lord R. G. Foti R. J. de Vader C. L. (1984). A test of leadership categorization theory: Internal structure, information processing, and leadership perceptions. Organizational Behavior and Human Performance, 34(3), 343–378. 10.1016/0030-5073(84)90043-6

[bibr73-01461672241246388] MacKinnon D. P. Krull J. L. Lockwood C. M. (2000). Equivalence of the mediation, confounding and suppression effect. Prevention Science, 1(4), 173–181. 10.1023/A:1026595011371 11523746 PMC2819361

[bibr74-01461672241246388] Marinova S. v Moon H. Kamdar D. (2012). Getting ahead or getting along? The two-facet conceptualization of conscientiousness and leadership emergence. Organization Science, 24(4), 1257–1276. 10.1287/orsc.1120.0781

[bibr75-01461672241246388] McCrae R. R. John O. P. (1992). An introduction to the five-factor model and its applications. Journal of Personality, 60(2), 175–215. 10.1111/j.1467-6494.1992.tb00970.x 1635039

[bibr76-01461672241246388] Meindl J. R. (1995). The romance of leadership as a follower-centric theory: A social constructionist approach. The Leadership Quarterly, 6(3), 329–341. 10.1016/1048-9843(95)90012-8

[bibr77-01461672241246388] Naumann L. P. Vazire S. Rentfrow P. J. Gosling S. D. (2009). Personality judgments based on physical appearance. Personality and Social Psychology Bulletin, 35(12), 1661–1671. 10.1177/0146167209346309 19762717

[bibr78-01461672241246388] Nemiroff P. M. Pasmore W. A. (2008). Lost at sea: A consensus-seeking task. In Biech E. (Ed.), The Pfeiffer book of successful team-building tools: Best of the annuals (2nd ed., pp. 165–172). Pfeiffer.

[bibr79-01461672241246388] Nestler S. Back M. D. (2013). Applications and extensions of the lens model to understand interpersonal judgments at zero acquaintance. Current Directions in Psychological Science, 22(5), 374–379. 10.1177/0963721413486148

[bibr80-01461672241246388] Offermann L. R. Kennedy J. K. Wirtz P. W. (1994). Implicit leadership theories: Content, structure, and generalizability. The Leadership Quarterly, 5(1), 43–58. 10.1016/1048-9843(94)90005-1

[bibr81-01461672241246388] Ong C. W. Roberts R. Arthur C. A. Woodman T. Akehurst S. (2016). The leader ship is sinking: A temporal investigation of narcissistic leadership. Journal of Personality, 84(2), 237–247. 10.1111/jopy.12155 25487857

[bibr82-01461672241246388] Ormiston M. E. Wong E. M. Ha J. (2022). The role of CEO emotional stability and team heterogeneity in shaping the top management team affective tone and firm performance relationship. The Leadership Quarterly, 33(3), 101543. 10.1016/j.leaqua.2021.101543

[bibr83-01461672241246388] Preacher K. J. Hayes A. F. (2008). Asymptotic and resampling strategies for assessing and comparing indirect effects in multiple mediator models. Behavior Research Methods, 40(3), 879–891. 10.3758/BRM.40.3.879 18697684

[bibr84-01461672241246388] Razinskas S. Hartmann S. (2023, June 14–16). A literature review on the role of psychological resilience in leadership contexts [Conference presentation]. The 23rd Annual Meeting of the European Academy of Management, Dublin, Ireland.

[bibr85-01461672241246388] Reichard R. J. Riggio R. E. Guerin D. W. Oliver P. H. Gottfried A. W. Gottfried A. E. (2011). A longitudinal analysis of relationships between adolescent personality and intelligence with adult leader emergence and transformational leadership. The Leadership Quarterly, 22(3), 471–481. 10.1016/j.leaqua.2011.04.005

[bibr86-01461672241246388] Robins R. W. Beer J. S. (2001). Positive illusions about the self: Short-term benefits and long-term costs. Journal of Personality and Social Psychology, 80(2), 340–352. 10.1037/0022-3514.80.2.340 11220450

[bibr87-01461672241246388] Schoemann A. M. Boulton A. J. Short S. D. (2017). Determining power and sample size for simple and complex mediation models. Social Psychological and Personality Science, 8(4), 379–386. 10.1177/1948550617715068

[bibr88-01461672241246388] Schönbrodt F. D. Back M. D. Schmukle S. C. (2012). TripleR: An R package for social relations analyses based on round-robin designs. Behavior Research Methods, 44(2), 455–470. 10.3758/s13428-011-0150-4 21909865

[bibr89-01461672241246388] Silard A. Dasborough M. T. (2021). Beyond emotion valence and arousal: A new focus on the target of leader emotion expression within leader–member dyads. Journal of Organizational Behavior, 42(9), 1186–1201. 10.1002/job.2513

[bibr90-01461672241246388] Soto C. J. John O. P. (2017). The next Big Five Inventory (BFI-2): Developing and assessing a hierarchical model with 15 facets to enhance bandwidth, fidelity, and predictive power. Journal of Personality and Social Psychology, 113(1), 117–143. 10.1037/pspp0000096 27055049

[bibr91-01461672241246388] Stangor C. (2015). Social groups in action and interaction (2nd ed.). Routledge. 10.4324/9780203338667

[bibr92-01461672241246388] Stavrova O. Evans A. M. van Beest I. (2023). The effects of partner extraversion and agreeableness on trust. Personality and Social Psychology Bulletin, 49(7), 1028–1042. 10.1177/01461672221086768 35481439 PMC10302358

[bibr93-01461672241246388] Tett R. P. Guterman H. A. (2000). Situation trait relevance, trait expression, and cross-situational consistency: Testing a principle of trait activation. Journal of Research in Personality, 34(4), 397–423. 10.1006/jrpe.2000.2292

[bibr94-01461672241246388] Van Fleet D. D. Atwater L. (1997). Gender neutral names: Don’t be so sure!. Sex Roles, 37(1–2), 111–123. 10.1023/A:1025696905342/METRICS

[bibr95-01461672241246388] Wiggins J. S. (1979). A psychological taxonomy of trait-descriptive terms: The interpersonal domain. Journal of Personality and Social Psychology, 37(3), 395–412. 10.1037/0022-3514.37.3.395

[bibr96-01461672241246388] Williams E. J. (1959). The comparison of regression variables. Journal of the Royal Statistical Society: Series B (Methodological), 21(2), 396–399. 10.1111/j.2517-6161.1959.tb00346.x

[bibr97-01461672241246388] Witkower Z. Tracy J. L. Cheng J. T. Henrich J. (2020). Two signals of social rank: Prestige and dominance are associated with distinct nonverbal displays. Journal of Personality and Social Psychology, 118(1), 89–120. 10.1037/pspi0000181 31021104

[bibr98-01461672241246388] Wojciszke B. Abele A. E. Baryla W. (2009). Two dimensions of interpersonal attitudes: Liking depends on communion, respect depends on agency. European Journal of Social Psychology, 39(6), 973–990. 10.1002/ejsp.595

[bibr99-01461672241246388] Wrzus C. Mehl M. R. (2015). Lab and/or field? Measuring personality processes and their social consequences. European Journal of Personality, 29(2), 250–271. 10.1002/per.1986

[bibr100-01461672241246388] Yukl G. (2008). How leaders influence organizational effectiveness. The Leadership Quarterly, 19(6), 708–722. 10.1016/j.leaqua.2008.09.008

[bibr101-01461672241246388] Yukl G. (2012). Effective leadership behavior: What we know and what questions need more attention. Academy of Management Perspectives, 26(4), 66–85. 10.5465/amp.2012.0088

[bibr102-01461672241246388] Yukl G. Gordon A. Taber T. (2002). A hierarchical taxonomy of leadership behavior: Integrating a half century of behavior research. Journal of Leadership & Organizational Studies, 9(1), 15–32. 10.1177/107179190200900102

[bibr103-01461672241246388] Yukl G. O’Donnell M. Taber T. (2009). Influence of leader behaviors on the leader-member exchange relationship. Journal of Managerial Psychology, 24(4), 289–299. 10.1108/02683940910952697

[bibr104-01461672241246388] Zaccaro S. J. Green J. P. Dubrow S. Kolze M. (2018). Leader individual differences, situational parameters, and leadership outcomes: A comprehensive review and integration. The Leadership Quarterly, 29(1), 2–43. 10.1016/j.leaqua.2017.10.003

[bibr105-01461672241246388] Zaccaro S. J. Rittman A. L. Marks M. A. (2001). Team leadership. The Leadership Quarterly, 12(4), 451–483. 10.1016/S1048-9843(01)00093-5

