# Supplementary material for: Differential Behavioral Pathways Linking Personality to Leadership Emergence and Effectiveness in Groups
Source: Pers Soc Psychol Bull. 2024 Apr 24;51(11):2166–82. doi: 10.1177/01461672241246388 (PMC12446710; doi:10.1177/01461672241246388)
Supplement: sj-docx-1-psp-10.1177_01461672241246388 – Supplemental material for Differential Behavioral Pathways Linking Personality to Leadership Emergence and Effectiveness in Groups [file sj-docx-1-psp-10.1177_01461672241246388.docx]

**Differential Behavioral Pathways Linking Personality to Leadership Emergence and Effectiveness in Groups**

**Supplemental Results**

Supplement A

Results of MMMs Controlled for Round, Gender, and Group Performance

**Figure A1**

*Model Results of the Behavioral Pathways Linking Personality to Leadership Outcomes Controlled for Round*

**
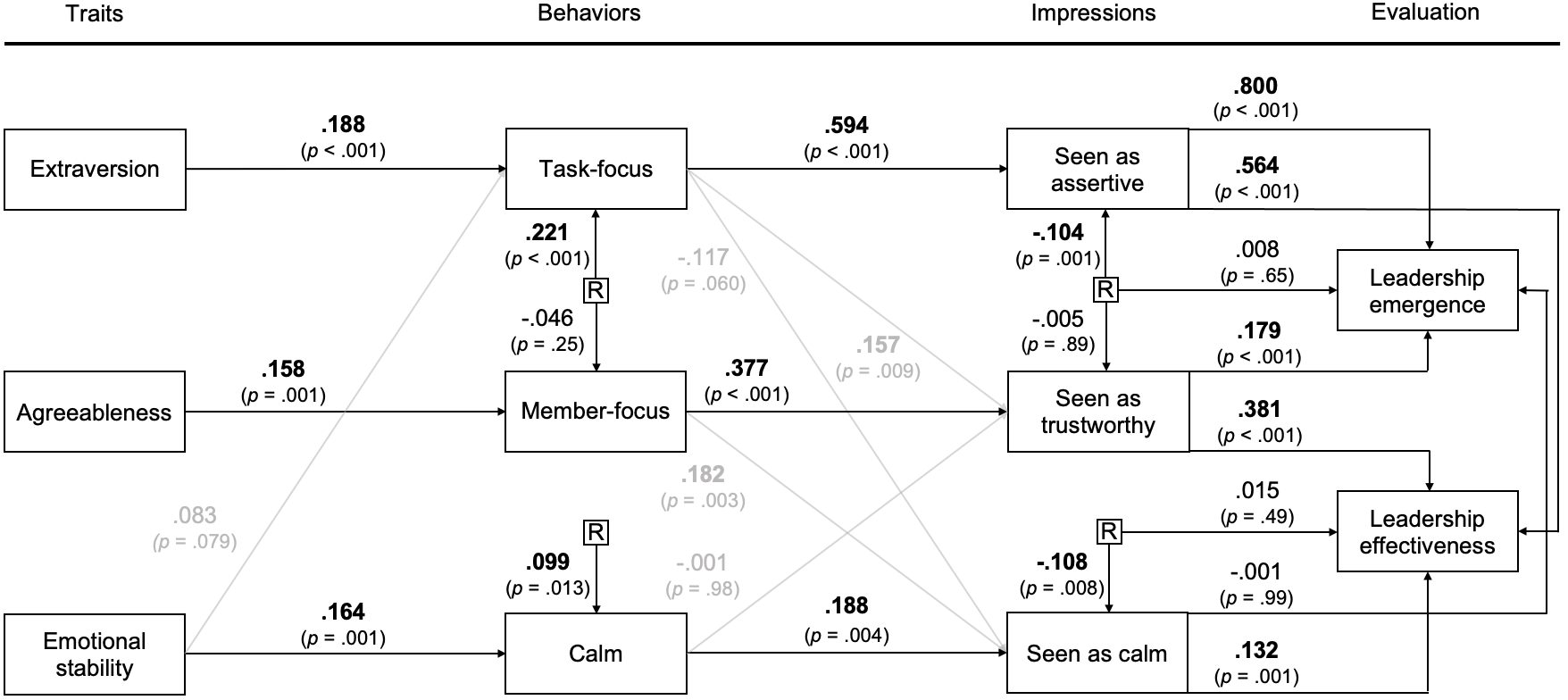
**

*Note*. R = Round in which the leader led the group (1-5). Personality traits, expressed behaviors, interpersonal impressions, and evaluations were allowed to covary among each other (paths are not displayed for the sake of clarity). Results are presented as standardized path coefficients.

Standardized path coefficients in bold were significant at the *p* < .05 level.

**Table A1**

*Model Fit, Direct and Indirect Effects of Personality Traits on Leadership Outcomes Controlled for Round*

|  | Leadership emergence | | | | | Leadership effectiveness | | | | | ∆IE | | |
| --- | --- | --- | --- | --- | --- | --- | --- | --- | --- | --- | --- | --- | --- |
|  |  |  |  | 95% CI | |  |  |  | 95% CI | |  | 95% CI | |
|  | DE | *p*_DE_ | IE | LL_IE_ | UL_IE_ | DE | *p*_DE_ | IE | LL_IE_ | UL_IE_ | IE | LL_IE_ | UL_IE_ |
| 1. Extraversion | **.055** | .029 | **.089** | .039 | .144 | **.090** | .004 | **.063** | .027 | .102 | **-.026** | -.045 | -.011 |
| 2. Agreeableness | .014 | .60 | **.011** | .003 | .021 | .010 | .78 | **.023** | .008 | .043 | **.012** | .004 | .023 |
| 3. Emotional stability | .041 | .14 | .000 | -.003 | .002 | .036 | .30 | **.004** | .001 | .010 | **.004** | .001 | .010 |
| χ²(25) | **86.2** |  |  |  |  |  |  |  |  |  |  |  |  |
| *p*_χ²_ | < .001 |  |  |  |  |  |  |  |  |  |  |  |  |
| RMSEA | .082 |  |  |  |  |  |  |  |  |  |  |  |  |
| SRMR | .032 |  |  |  |  |  |  |  |  |  |  |  |  |
| CFI | .966 |  |  |  |  |  |  |  |  |  |  |  |  |
| TLI | .919 |  |  |  |  |  |  |  |  |  |  |  |  |

*Note.* DE = direct effect, IE = indirect effect, CI = confidence interval, LL = lower limit, UL = upper limit.

DEs and IEs printed in bold are significant at the *p* < .05 level.

**Figure A2**

*Model Results of the Behavioral Pathways Linking Personality to Leadership Outcomes Controlled for Gender*

**
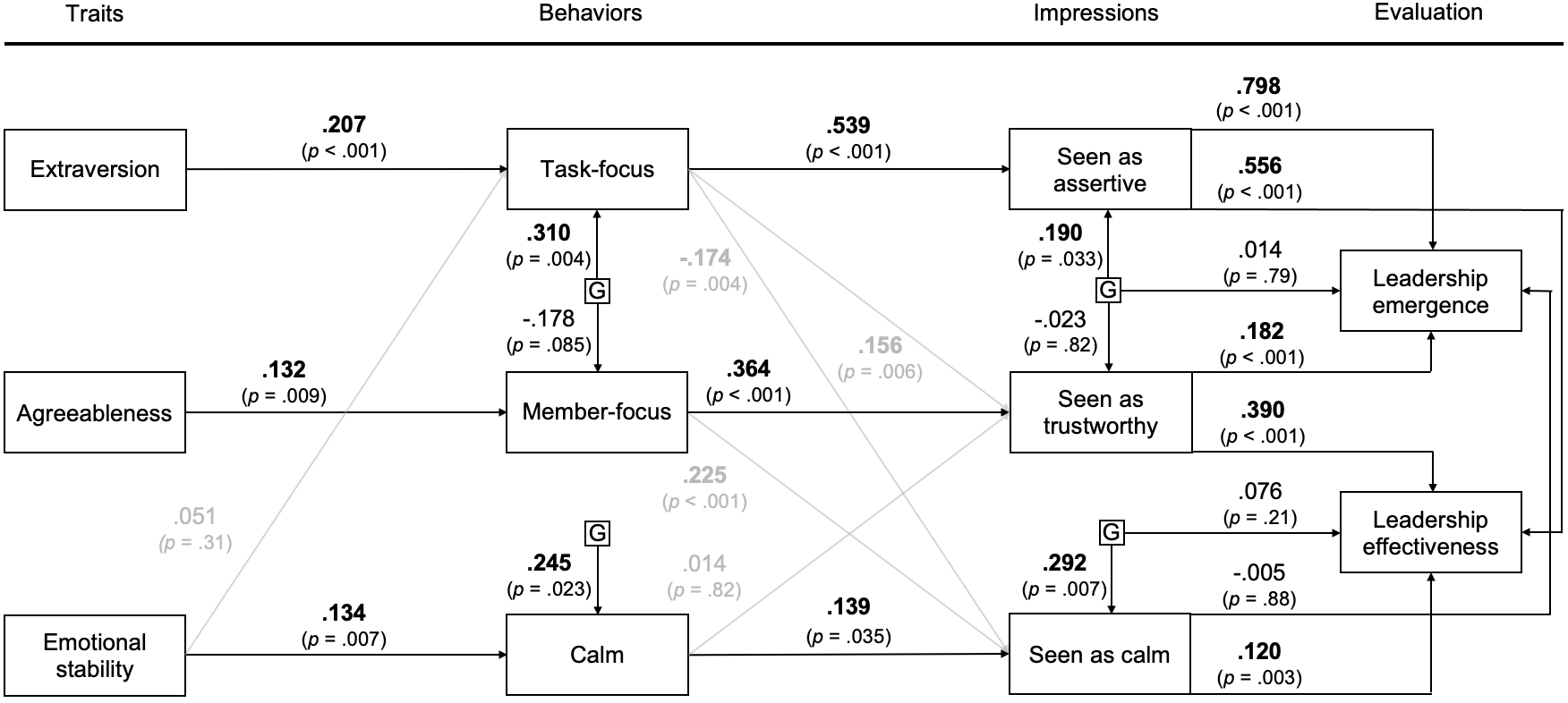
**

*Note*. G = Leader gender (0/1 = female/male). Personality traits, expressed behaviors, interpersonal impressions, and evaluations were allowed to covary among each other (paths are not displayed for the sake of clarity). Results are presented as standardized path coefficients.

Standardized path coefficients in bold were significant at the *p* < .05 level.

**Table A2**

*Model Fit, Direct and Indirect Effects of Personality Traits on Leadership Outcomes Controlled for Gender*

|  | Leadership emergence | | | | | Leadership effectiveness | | | | | ∆IE | | |
| --- | --- | --- | --- | --- | --- | --- | --- | --- | --- | --- | --- | --- | --- |
|  |  |  |  | 95% CI | |  |  |  | 95% CI | |  | 95% CI | |
|  | DE | *p*_DE_ | IE | LL_IE_ | UL_IE_ | DE | *p*_DE_ | IE | LL_IE_ | UL_IE_ | IE | LL_IE_ | UL_IE_ |
| 1. Extraversion | **.056** | .028 | **.089** | .041 | .143 | **.096** | .002 | **.062** | .028 | .101 | **-.027** | -.046 | -.012 |
| 2. Agreeableness | .015 | .58 | **.009** | .002 | .019 | .016 | .65 | **.019** | .004 | .038 | **.010** | .002 | .020 |
| 3. Emotional stability | .040 | .16 | .000 | -.002 | .001 | .028 | .44 | **.002** | .000 | .006 | **.002** | .000 | .007 |
| χ²(25) | **110.1** |  |  |  |  |  |  |  |  |  |  |  |  |
| *p*_χ²_ | < .001 |  |  |  |  |  |  |  |  |  |  |  |  |
| RMSEA | .097 |  |  |  |  |  |  |  |  |  |  |  |  |
| SRMR | .040 |  |  |  |  |  |  |  |  |  |  |  |  |
| CFI | .952 |  |  |  |  |  |  |  |  |  |  |  |  |
| TLI | .885 |  |  |  |  |  |  |  |  |  |  |  |  |

*Note.* DE = direct effect, IE = indirect effect, CI = confidence interval, LL = lower limit, UL = upper limit.

DEs and IEs printed in bold are significant at the *p* < .05 level.

**Figure A3**

*Model Results of the Behavioral Pathways Linking Personality to Leadership Outcomes Controlled for Group Performance*

**
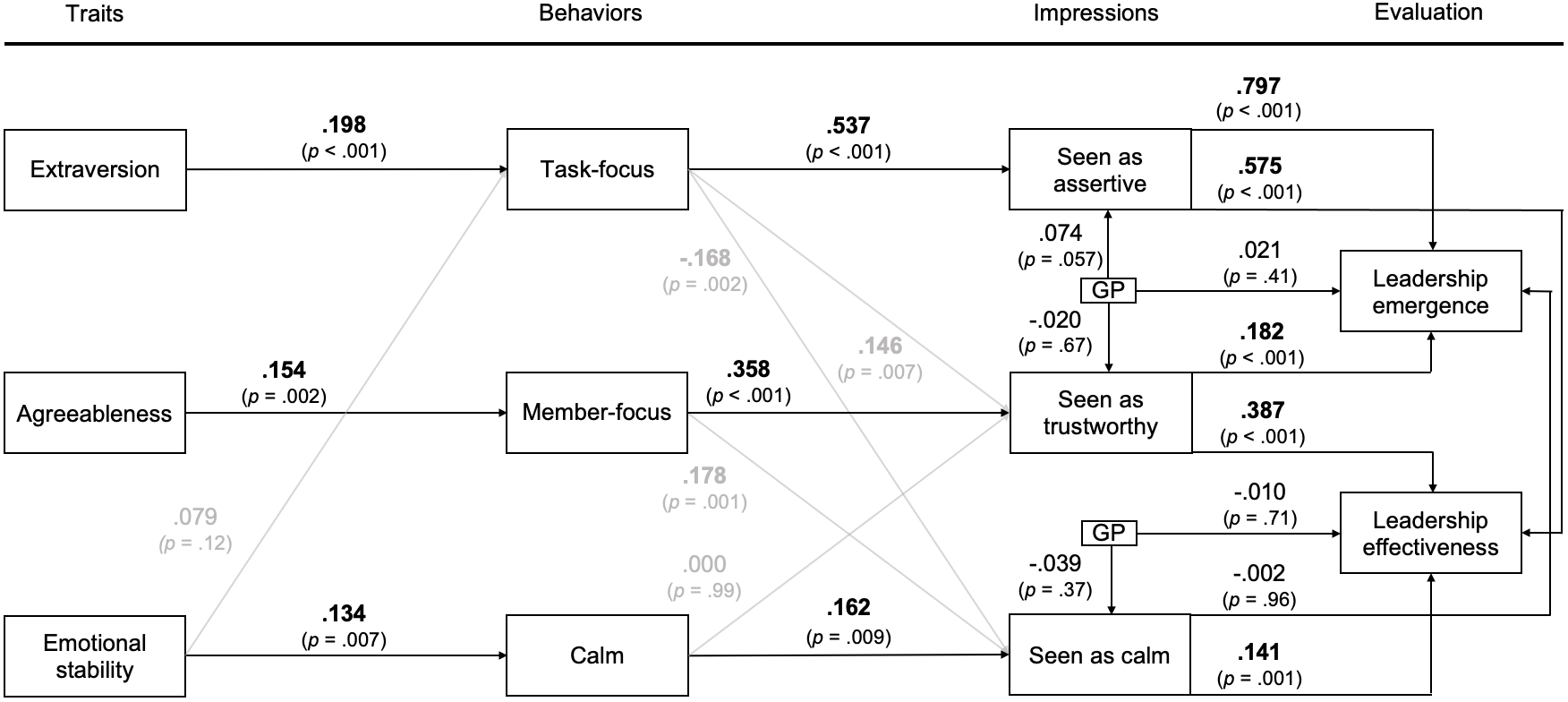
**

*Note*. GP = Group performance (higher values on this variable indicate larger deviations of group rankings from expert rankings and thus lower performance). Personality traits, expressed behaviors, interpersonal impressions, and evaluations were allowed to covary among each other (paths are not displayed for the sake of clarity). Results are presented as standardized path coefficients.

Standardized path coefficients in bold were significant at the *p* < .05 level.

**Table A3**

*Model Fit, Direct and Indirect Effects of Personality Traits on Leadership Outcomes Controlled for Group Performance*

|  | Leadership emergence | | | | | Leadership effectiveness | | | | | ∆IE | | |
| --- | --- | --- | --- | --- | --- | --- | --- | --- | --- | --- | --- | --- | --- |
|  |  |  |  | 95% CI | |  |  |  | 95% CI | |  | 95% CI | |
|  | DE | *p*_DE_ | IE | LL_IE_ | UL_IE_ | DE | *p*_DE_ | IE | LL_IE_ | UL_IE_ | IE | LL_IE_ | UL_IE_ |
| 1. Extraversion | **.055** | .030 | **.085** | .037 | .137 | **.089** | .005 | **.061** | .027 | .101 | **-.024** | -.040 | -.010 |
| 2. Agreeableness | .014 | .62 | **.010** | .003 | .021 | .015 | .67 | **.021** | .007 | .041 | **.011** | .004 | .022 |
| 3. Emotional stability | .039 | .17 | .000 | -.002 | .002 | .035 | .32 | **.004** | .000 | .009 | **.004** | .001 | .009 |
| χ²(28) | **96.9** |  |  |  |  |  |  |  |  |  |  |  |  |
| *p*_χ²_ | < .001 |  |  |  |  |  |  |  |  |  |  |  |  |
| RMSEA | .082 |  |  |  |  |  |  |  |  |  |  |  |  |
| SRMR | .039 |  |  |  |  |  |  |  |  |  |  |  |  |
| CFI | .960 |  |  |  |  |  |  |  |  |  |  |  |  |
| TLI | .913 |  |  |  |  |  |  |  |  |  |  |  |  |

*Note.* DE = direct effect, IE = indirect effect, CI = confidence interval, LL = lower limit, UL = upper limit.

DEs and IEs printed in bold are significant at the *p* < .05 level.
